# Detrimental Effects of Filling Laryngotracheal Airways To Excessive Pressure (DEFLATE-P): a quality improvement initiative

**DOI:** 10.1186/s12871-020-00963-6

**Published:** 2020-02-24

**Authors:** Ashley V. Fritz, Gregory J. Mickus, Michael A. Vega, J. Ross Renew, Sorin J. Brull

**Affiliations:** Department of Anesthesiology and Perioperative Medicine, Mayo Clinic, 4500 San Pablo Road S, Jacksonville, Florida 32224 USA

**Keywords:** Sore throat, Cough, Vocal cord damage, Cuff pressure, Manometry

## Abstract

**Background:**

This quality improvement (QI) project was performed at a single center to determine the incidence of postoperative complications associated with use of cuffed airway devices. An educational program was then completed that involved training our anesthesia providers about complications related to excessive cuff pressure and how to utilize a quantitative cuff pressure measurement device (manometer). The impact of this educational initiative was assessed by comparing the incidence of postoperative complications associated with the use of airway devices before and after the training period.

**Methods:**

After approval by our institution’s Institutional Review Board, a pre-intervention (baseline) survey was obtained from 259 adult patients after having undergone surgery with general anesthesia with the use of an endotracheal tube (ETT) or laryngeal mask airway (LMA). Survey responses were used to determine the baseline incidence of sore throat, hoarseness, and dysphagia. Once these results were obtained, education was provided to the anesthesia department members addressing the complications associated with excessive cuff pressures, appropriate cuff pressures based on manufacturer recommendations, and instructions on the use of a quantitative monitor to determine cuff pressure (manometry). Clinical care was then changed by requiring intraoperative cuff pressure monitoring throughout our institution for all surgical patients. After this educational period, 299 patients completed the same survey describing postoperative airway complications.

**Results:**

The use of manometry reduced the incidence of moderate-to-severe postoperative sore throat in the pre- vs. post-intervention groups (35 patients vs 31 patients, *p* = 0.045), moderate to severe hoarseness (30 patients vs 13, patients *p* = 0.0001), and moderate-to-severe dysphagia (13 patients vs 5 patients, *p* = 0.03).

**Conclusion:**

Caring for patients in the perioperative setting frequently entails placement of an airway device. This procedure is associated with several potential complications, including sore throat, coughing, and vocal cord damage. Our quality improvement initiative has shown that intraoperative management of intra-cuff pressure based on manometry is feasible to implement in clinical practice and can reduce postoperative airway complications.

## Background

Sore throat, dysphagia, and hoarseness are multifactorial postoperative complications after general anesthesia in which manipulation of the airway is required. The incidence of post-operative sore throat (POST) has been reported to occur in 17.5–26% of postoperative patients [1, 2] but some studies have reported incidences as high as 50% [3, 4]. The pathologic process has been suggested to involve direct mucosal injury and inflammation related to airway instrumentation and presence of foreign airway objects [5]. Excessive cuff pressures of airway devices have been implicated as a cause of POST [6, 7].

There are reports of mixed efficacy in prevention of POST by pharmacologic modalities, such as topical anesthetics [8]. Moreover, non-pharmacologic modalities, including preoperative licorice water gargling, [9] have also been reviewed, ultimately showing limited impact on complications [5, 7, 9]. Alternatively, Farhang et al. showed that preoperative zinc lozenges were able to reduce the incidence of POST by 24% (*p* < .05) within the first 2 post-operative hours [10].

Chang et al. demonstrated that tapered ETT cuffs, as compared to cylindrical ones, reduce post-operative sore throat by as much as 22% (*p* = .003) [11]. Interestingly, two studies comparing open abdominal surgery to laparoscopic techniques revealed that manometry-measured endotracheal tube (ETT) cuff pressures significantly increased after abdominal insufflation or Trendelenburg positioning, and subsequently resulted in more frequent POST events [12, 13]. Koyama et al. demonstrated that the application of lubricant to the cuff can prevent such increases in pressure via a reduction of gas diffusion into the cuff [14]. Another study compared a novel laryngeal mask airway (LMA) with intra-cuff pressure measurement versus a traditional LMA, finding a significant reduction in postoperative pharyngolaryngeal complications [15]. Interestingly, Corda et al. found that utilizing syringe rebound pressure alone (as a surrogate for measurement of intra-cuff pressure with manometry) was enough to reduce the incidence of POST [16]. In lieu of manometry, it is worthwhile to note two double-blind, randomized controlled trials that have reported success in reducing postoperative respiratory complications by titration of ETT cuff pressure based on the anesthesia machine volume-time curve and minimizing or eliminating the difference between the inspiratory and expiratory volume [17, 18]. None of these interventions, however, completely eliminated POST.

Most clinicians employ subjective measures to mitigate the burden of POST, such as manually palpating the pilot balloon to assess intra-cuff pressure. However, objective measurements of ETT cuff pressures to obtain and maintain recommended ranges provide a more significant reduction in these postoperative complications [6]. A review of several studies has recently introduced the idea of physical tissue damage as a precursor to postoperative airway complications [5], consistent with other recent studies regarding manometry [6, 7, 13, 19–21]. Minimizing airway cuff pressures may be the key to reducing POST. Although the use of manometry is not commonplace, the availability of new, compact and portable devices allows easy integration into everyday practice and may help reduce the incidence of postoperative complications [8, 19, 20]. We therefore initiated this quality improvement project to determine whether monitoring of cuff pressures (and presumably, preventing intraoperative cuff over inflation) would translate into improved patient care and a lower incidence of complications. We deliberately used a novel, easy to use, and inexpensive manometer (the AG CUFFILL) to facilitate provider acceptance and use. Other pressure monitors (for instance, Cufflator manometer, manufactured by Posey, Sulz, Germany) are much larger and more expensive, and need to be cleaned to prevent vertical bacterial transmission.

One of the most important etiologies of POST is tissue ischemia secondary to cuff over-distension. In our study, we sought to eliminate overinflated cuffs by measuring and ensuring appropriate intraoperative intra-cuff pressure. Our project aimed to determine the incidence of postoperative airway complications at our institution, and inform our anesthesia providers of these patient care concerns. We also sought to educate our department about airway complications that might be due to excessive airway device cuff pressures. Finally, we adjusted clinical practice to incorporate routine measurement of cuff pressures after airway manipulation and then investigated whether this practice change had an impact on the incidence and severity of POST.

## Methods

After receiving Institutional Review Board (IRB) approval, a pre-intervention baseline assessment of POST complications was conducted through an originally developed questionnaire (Additional File 1) that documented the airway device utilized, dexamethasone administration, and contained questions delineating the severity of sore throat, hoarseness, coughing, and dysphagia in the postoperative period (Supplemental Material). The symptoms were rated on a 4-point subjective, verbal scale ranging from “none” to “severe.” A questionnaire was administered to adult patients (> 18 years old) who had undergone outpatient or inpatient surgery under general anesthesia with ETT or LMA. The questionnaires were completed prior to discharge from Phase 2 recovery. The discharge criteria are based on Aldrete’s Modified Phase I Post-anesthesia Recovery Score, as depicted in Table 1 [22]. No patients were excluded from participation in this post-operative assessment after the practice change was implemented, and pre-intervention questionnaires were collected over several months at random intervals. A power analysis was performed using the lowest reported incidence of POST (14.4%), considering a 50% reduction in the incidence as significant at *p* < 0.05. To reach statistical significance, a total of 200 patients (*n* = 200) were considered for inclusion. Of note, during the pre-intervention time period, physicians and nurse anesthetists were blinded to the project intent.

**Table 1:** Aldrete’s Modified Phase I Post Anesthesia Recovery Score

Neurologic	• Fully awake, able to answer questions • Able to move four extremities voluntarily or on command
Respiratory	• Breathing deeply and coughing freely • Able to maintain oxygen saturation > 92% on room air
Cardiovascular	• Blood pressure and heart rate within 20% of pre-anesthetic/sedation level

**Fig. 1:**
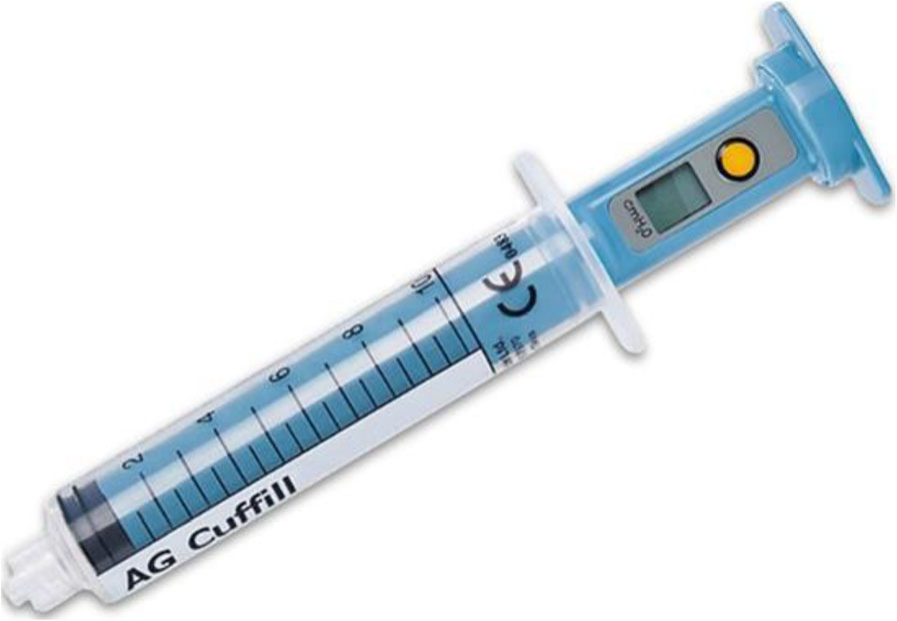
AG Cuffiill™ Manometry Device. The manometry device (cmH2O) provided for cuff pressure measurement during the post-interventiom phase

An educational module was created and distributed electronically to our anesthesia providers. The module contained pre-test questions and evaluated knowledge of the incidence of POST as well as the recommended ETT and LMA cuff pressures. Information on the reported incidence of POST and recommended cuff pressures was provided, as well as step-by-step text instructions for measurement of cuff pressure with an electronic manometry device (AG CUFFILL™), (Hospitech Respiration, Ltd., Mercury Medical, Clearwater, FL) (Fig. 1). This in-service was followed by a brief instructional video and a link to the manufacturer’s instructional video. Changes were made to the electronic medical record (EMR) prompting provider input of ETT and LMA cuff pressures before and after intervention. This built-in visual reminder assisted the transition of objective manometry assessment into everyday practice.

The post-intervention questionnaire was collected over 10 months, and contained questions identical to the pre-intervention questionnaire with additional fields for initial and adjusted cuff pressures, where applicable. Data analysis was performed using a two-tailed Fisher’s exact test to determine the impact our educational intervention on initial cuff pressures, adjusted cuff pressures, as well as the frequency of POST, hoarseness, and dysphagia.

## Results

**Fig. 2:**
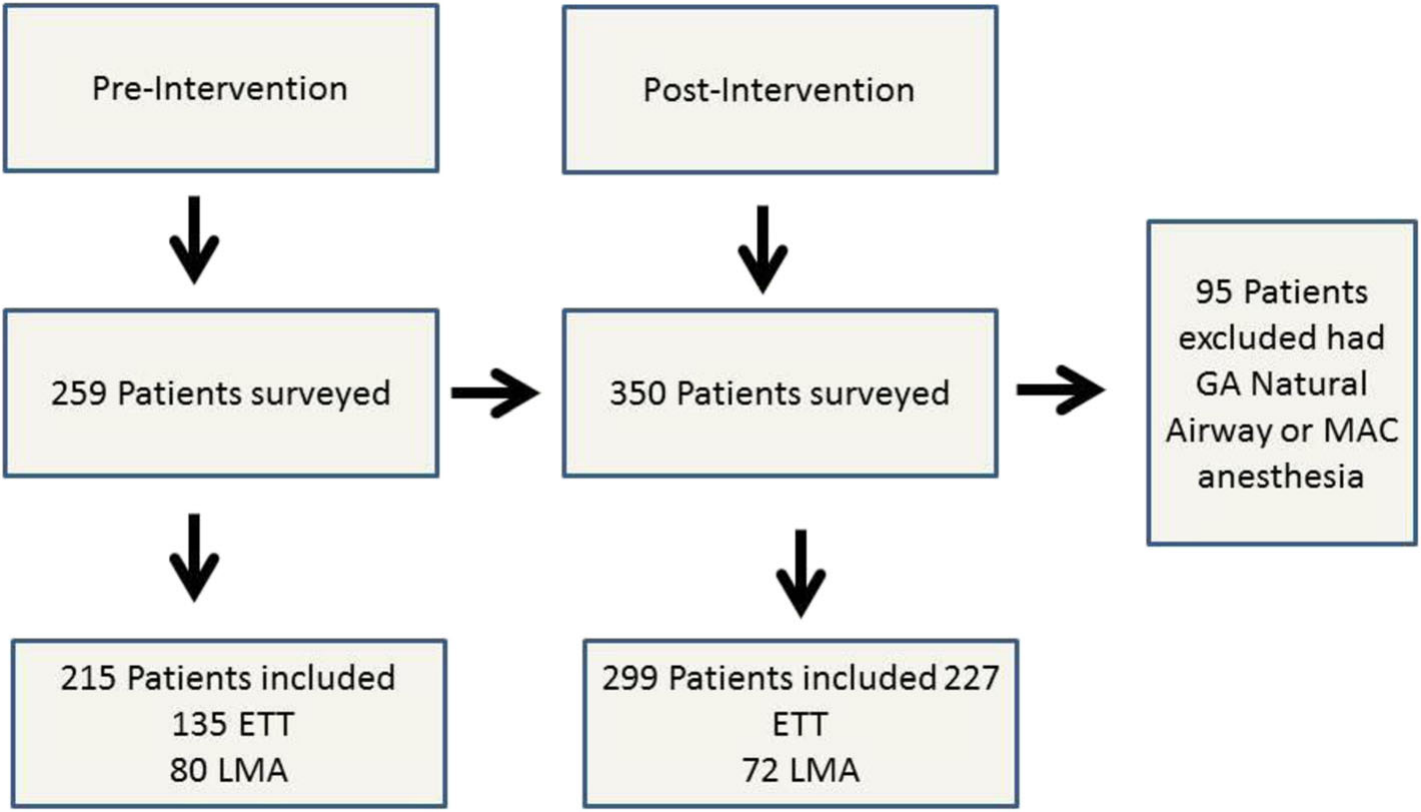
Project inclusion criteria Flowchart. Pre and post intervention patients surveyed, included, and excluded from quality improvement project

A total of 259 pre-intervention and 350 post-intervention questionnaires were collected over 23 months, including 362 ETTs and 152 LMAs (Fig. 2). Subsequently, 95 patients were excluded secondary to undergoing general anesthesia with a natural airway or monitored anesthesia care (MAC). The use of manometry reduced the incidence of moderate-severe postoperative sore throat in the pre- and post-intervention groups (35 patients vs 31 patients, *p* = 0.045, respectively). Moderate to severe hoarseness was also reduced with the application of manometry when comparing pre-intervention (initial pressure) and post-intervention (adjusted pressure) groups (30 patients vs 13 patients, *p* = 0.0001), respectively. Finally, moderate to severe dysphagia was also reduced with the use of manometry when comparing pre- and post- intervention groups (13 patients vs 5 patients, *p* = 0.03, respectively), as identified in Table 2. The use of manometry demonstrated that initial cuff pressures in both airway devices were consistently above recommended values. On average, the initial ETT cuff pressure was 38 ± 18 cm H_2_O. Average adjusted pressure for ETTs was 27 ± 4 cm H_2_O. Mean initial pressure for LMAs was 66 ± 18 cm H_2_O. Mean adjusted pressure for LMAs was 55 ± 8.4 cm H_2_O (Table 3).

**Table 2:** Reduction in Postoperative Airway Complications

*N* = 299 total	Pre-Intervention (n)	Post-Intervention (n)	*P*-Value
Sore Throat	35	31	0.0457
Hoarseness	30	13	0.0001
Dysphagia	13	5	0.0301

**Table 3:** Post-intervention initial and adjusted cuff pressures (cm H2O)

*N* = 299 total	Initial (SD)	Adjusted (SD)	Range Pre-Intervention	Range Post-Intervention
ETT	38 (18)	26.7 (4.3)	9 to OP	17 to 30
LMA	66.1 (17.7)	54.8 (8.4)	20 to OP	22 to 60

*OP* Over Pressure or beyond 100 cm H2O, *ETT* Endotracheal Tube, *LMA* Laryngeal Mask Airway, *SD* Standard Deviation

## Discussion

This QI project demonstrated that the utilization of a quantitative device that objectively determines the pressures of ETT and LMA cuffs can reduce the incidence of postoperative airway complications. Specifically, this action led to a significant reduction in the incidence of sore throat (*p* = 0.045), hoarseness (*p* = < 0.001), and dysphagia (*p* = 0.03) (Table 2).

Our findings were in congruence with previous studies. Liu et al. evaluated 509 patients undergoing elective surgery with general anesthesia and endotracheal intubation. These investigators determined that patients who had their cuff pressures adjusted had a lower incidence of post-operative sore throat (*p* = < 0.001) as well as decreased injury to tracheal mucosa as indicated by blood streaked expectoration (*p* = 0.089) [6]. A similar study conducted by Li et al. evaluated the incidence of post-operative respiratory complications in patients who underwent general anesthesia with LMAs and demonstrated lower LMA cuff pressures decreased incidence of sore throat (*p* = 0.022), and dysphagia (*p* = 0.007) [23].

While it is difficult to quantify the total cost of postoperative airway complications, the cost of the intervention is minimal. For instance, the AG CUFFILL device can be reused up to 100 times, since measurement of cuff pressures needs not be performed in a sterile fashion. The AG CUFFILL syringe, for instance, has a local cost of $20.00 per unit, resulting in a “cost per use” of only $0.20. The cost of reusable manometers such as the Cufflator™ (VBM Cuff Pressure Gauge, VBM Medizintechnik GmbH, Sulz, Germany) and the Portex Cuff Inflator Pressure Gauge (Smith Medical ASD Inc., Dublin, OH, USA) require a more substantial initial investment of $309.00 and $84.95, respectively. These devices are also larger than the AG CUFFILL syringe, making their use and storage in the limited OR setting much more problematic. These devices also need to be calibrated regularly and require routine maintenance, increasing their cost of use. In comparison, the AG CUFFILL device does not require routine calibration. While it is difficult to quantify the financial burden of postoperative airway complications, patient safety and satisfaction are universally paramount. Future endeavors describing the effects of confounders may help develop more appropriate and simpler methods to alleviate and prevent postoperative airway complications.

There are several limitations to our project. Manometry represents one objective measure to reduce postoperative respiratory complications; however this is a dilemma with a multitude of variables. Other factors not evaluated in our project that can contribute to POST include preoperative sore throat, trauma during instrumentation of a difficult airway, size of the airway device, the use of neuromuscular blocking agents, presence/absence of cuff lubrication, and length of surgery. Our project may have also been influenced by the Hawthorne Effect as providers knew that cuff pressures were going to be checked after our educational intervention.

As with any new endeavor, there will be challenges to implementation and resistance to alteration of current practice. Our initiative provides insight into such a process, with emphasis on brief, straight-forward education. With the marked reduction in unadjusted and adjusted cuff pressures during the post-intervention phase, our results reinforce how common overinflated cuff pressures are and establishes that utilizing a quantitative device to guide cuff pressure is feasible. In addition, the EMR (electronic medical record) can provide visual cues and reminders to the provider requiring the clinician to measure, adjust, and document the ETT and LMA cuff pressures. The online module, instructional videos, hands-on tutorial, as well as EMR reinforcement solidified the behavioral modification for cuff pressure monitoring. Future investigations are warranted to confirm such findings.

## Conclusions

Caring for patients in the perioperative setting often entails placement of an airway device with potential adverse effects and complications that warrant further investigation. Our quality initiative has shown it is feasible to implement manometry into everyday practice, which resulted in a marked reduction in postoperative airway complications. As technology continues to advance, it is imperative for us to utilize the tools provided to our advantage, for the benefit of our patients, and the establishment of a refined standard of care.

## Supplementary information

**Additional file 1.** Postoperative Pharyngeal Assessment Questionnaire This is an internally developed questionnaire by our institution to assess the severity of sore throat, dysphagia, coughing and hoarseness of postoperative patients.

## Data Availability

All original datasets used and analyzed during the current quality improvement project are available from the corresponding author on reasonable request.

## Abbreviations

EMR: Electronic Medical Record
ETT: Endotracheal Tube
IRB: Institutional Review Board
LMA: Laryngeal Mask Airway
MAC: Monitored Anesthesia Care
OP: Over Pressure
POST: Post-Operative Sore Throat
QI: Quality Improvement
SD: Standard Deviation
