# Supplementary material for: Detrimental Effects of Filling Laryngotracheal Airways To Excessive Pressure (DEFLATE-P): a quality improvement initiative
Source: BMC Anesthesiol. 2020 Feb 24;20:46. doi: 10.1186/s12871-020-00963-6 (PMC7038516; doi:10.1186/s12871-020-00963-6)
Supplement: Supplementary file 1 — Additional file 1. Postoperative Pharyngeal Assessment Questionnaire This is an internally developed questionnaire by our institution to assess the severity of sore throat, dysphagia, coughing and hoarseness of postoperative patients. [file 12871_2020_963_MOESM1_ESM.pdf]

# Postoperative pharyngolaryngeal assessment

Study ID ________________________

Date of surgery ________________________

What type of airway device was used intraoperatively?
- ☐ None
- ☐ Oral airway
- ☐ Nasal trumpet
- ☐ Endotracheal tube (ETT)
- ☐ Laryngeal Mask Airway (LMA)

Was dexamethasone administered?
- ☐ Yes
- ☐ No

---

**At the time of patient's discharge from Phase 2 recovery period, assess the following:**

Sore throat
- ☐ No sore throat or any throat discomfort
- ☐ Minimal sore throat
- ☐ Moderate sore throat
- ☐ Severe sore throat.

Hoarseness
- ☐ No hoarseness
- ☐ Mild
- ☐ Moderate
- ☐ Severe

Coughing
- ☐ none
- ☐ mild (less than a common cold)
- ☐ moderate (similar to a common cold)
- ☐ severe (more than a common cold)

Dysphagia
- ☐ none (no episode of swallowing difficulty)
- ☐ mild (experienced only rare episodes of dysphagia and not considred a significant problem)
- ☐ moderate (occasioanl swallowing difficulty)
- ☐ severe (frequent difficulty swallowing)
